# Prenatal Exposure to Valproic Acid Affects Microglia and Synaptic Ultrastructure in a Brain-Region-Specific Manner in Young-Adult Male Rats: Relevance to Autism Spectrum Disorders

**DOI:** 10.3390/ijms21103576

**Published:** 2020-05-18

**Authors:** Magdalena Gąssowska-Dobrowolska, Magdalena Cieślik, Grzegorz Arkadiusz Czapski, Henryk Jęśko, Małgorzata Frontczak-Baniewicz, Magdalena Gewartowska, Agnieszka Dominiak, Rafał Polowy, Robert Kuba Filipkowski, Lidia Babiec, Agata Adamczyk

**Affiliations:** 1 Department of Cellular Signalling, Mossakowski Medical Research Centre, Polish Academy of Sciences, Pawińskiego 5, 02-106 Warsaw, Poland; mcieslik@imdik.pan.pl (M.C.); gczapski@imdik.pan.pl (G.A.C.); hjesko@imdik.pan.pl (H.J.); lbabiec@imdik.pan.pl (L.B.); 2 Electron Microscopy Platform, Mossakowski Medical Research Centre, Polish Academy of Sciences, Pawińskiego 5, 02-106 Warsaw, Poland; mbaniewicz@imdik.pan.pl (M.F.-B.); mgewartowska@imdik.pan.pl (M.G.); 3 Department of Biochemistry and Pharmacogenomics, Faculty of Pharmacy, Medical University of Warsaw, Banacha 1B, 02-097 Warsaw, Poland; adominiak212@gmail.com; 4 Behavior and Metabolism Research Laboratory, Mossakowski Medical Research Centre, Polish Academy of Sciences, Pawińskiego 5 St, 02-106 Warsaw, Poland; rpolowy@imdik.pan.pl (R.P.); rfilipkowski@imdik.pan.pl (R.K.F.)

**Keywords:** synaptic ultrastructure, pre- and postsynaptic proteins, synaptopathology, oxidative stress, neuroinflammation, microglia, valproic acid (VPA), autism spectrum disorders (ASD)

## Abstract

Autism spectrum disorders (ASD) are a heterogeneous group of neurodevelopmental conditions categorized as synaptopathies. Environmental risk factors contribute to ASD aetiology. In particular, prenatal exposure to the anti-epileptic drug valproic acid (VPA) may increase the risk of autism. In the present study, we investigated the effect of prenatal exposure to VPA on the synaptic morphology and expression of key synaptic proteins in the hippocampus and cerebral cortex of young-adult male offspring. To characterize the VPA-induced autism model, behavioural outcomes, microglia-related neuroinflammation, and oxidative stress were analysed. Our data showed that prenatal exposure to VPA impaired communication in neonatal rats, reduced their exploratory activity, and led to anxiety-like and repetitive behaviours in the young-adult animals. VPA-induced pathological alterations in the ultrastructures of synapses accompanied by deregulation of key pre- and postsynaptic structural and functional proteins. Moreover, VPA exposure altered the redox status and expression of proinflammatory genes in a brain region-specific manner. The disruption of synaptic structure and plasticity may be the primary insult responsible for autism-related behaviour in the offspring. The vulnerability of specific synaptic proteins to the epigenetic effects of VPA may highlight the potential mechanisms by which prenatal VPA exposure generates behavioural changes.

## 1. Introduction

Autism spectrum disorders (ASD) are a heterogeneous group of neurodevelopmental syndromes characterized by deficits in verbal and nonverbal communication and social interactions, along with stereotypical repetitive behaviours, and narrowed interests [1,2]. ASD affects about 1% of the worldwide population, and boys are about four times more frequently diagnosed than girls [3,4,5].

While ASD share characteristic features at the behavioural level, the pathogenesis of autism has not yet been clearly elucidated, and its underlying causes are highly heterogeneous. It is suggested that, in addition to genetic predispositions, the epigenetic changes evoked by maternal stressors, infectious agents, drugs, and environmental factors during the early phase of life have a latent and long-term impact on brain function, induce various congenital malformations, and increase the risk of ASD in offspring [6,7].

Although genetic screening has identified hundreds of mutations and other genetic variations associated with ASD, recent evidence revealed that many of these variations are associated with genes encoding proteins that affect various aspects of synaptic functions [2,8,9]. A significant proportion of these genes encode proteins that are localized mainly in the post-synaptic terminals, such as cell adhesion molecules (neuroligins (Nlgns) (especially Nlgn3 and Nlgn4X) [10,11,12]), which mediate the formation and maintenance of synapses [13] and the best-characterized scaffolding proteins (post-synaptic density protein 95 (PSD95) [14,15], as well as SH3 and multiple ankyrin repeat domains protein (Shank) [16,17]), which are essential for maintaining trans-synaptic connections, synapse stabilization, and synaptogenesis [18,19]. Among the presynaptic proteins, the adhesive partners for neuroligins, neurexins (Nrxn), as well as synapsin (Syn1) [13,20,21,22] and synaptosomal-associated protein 25 (SNAP25) [3,23], were also demonstrated to be affected in individuals with ASD. This suggests that synapses are a possible site of autism origin. Defects in specific synaptic proteins may produce abnormalities in cellular neurotransmissions at the excitatory (E) and inhibitory (I) synapses, disrupting the (E/I) balance and affecting neural plasticity, which could be one of the fundamental causative factors underlying ASD pathology [20,24]. Neurotransmitter release that allows fast communication between neurons requires proper vesicle tethering, docking, priming, and, finally, fusion with the plasma membrane. These events are driven by the SNARE (soluble N-ethylmaleimide-sensitive factor (NSF) attachment protein (SNAP) receptor) complex. This molecular fusion machinery is formed by the vesicle-associated membrane proteins (*v*-SNARE), synaptobrevins 1 and 2 (VAMP1/2), and the proteins acting as plasma membrane anchors (*t*-SNARE), syntaxin 1 (Stx1) and SNAP25. The cross-interaction between these core *v*-SNARE and *t*-SNARE proteins seems to play a fundamental role in synaptic exocytosis, neurotransmission, and plasticity [25,26,27,28]. Other synaptic proteins, such as synapsin (Syn), synaptophysin (Syp), and synaptotagmin (Syt), interact with the SNARE complex, regulating it and participating in different steps of synaptic exocytosis [29]. Studies have shown that genes encoding these presynaptic proteins are implicated in different neurodevelopmental and neuropsychiatric disorders, including ASD, attention deficit hyperactivity disorder (ADHD), epilepsy, bipolar disorder (BPD), schizophrenia (SCZ), and major depressive disorder (MDD) [3,13,20,21,30].

Despite the numerous links between synaptic dysfunction and the pathology of ASD, the precise molecular mechanisms underlying the pathogenesis of autism are not fully elucidated. Although alterations in some synaptic proteins have already been demonstrated in experimental models of ASD, the level of key proteins responsible for synapse formation, structure correctness, synaptic transmission, and plasticity were not investigated. Above all, the structure of the synaptic endings has not been investigated so far.

Therefore, the main goal of our study was to investigate the ultrastructure of synaptic endings, as well as the expression of key synaptic proteins responsible for the maintenance of synaptic structures and neurotransmitter release in a rat model of environmentally triggered autism based on embryological exposure *in utero* to valproic acid (VPA). Pregnant Wistar rats received a single intraperitoneal injection of VPA (450 mg/kg of body weight) on gestational day 12.5, which, in humans, corresponds to early/middle foetal life. We studied the hippocampus and cerebral cortex, the brain areas that control many of the executive functions of the brain, including higher-order cognitive processes, such as decision-making, planning, working memory, emotions, social behaviour, learning, and communication.

## 2. Results

### 2.1. Prenatal Exposure to VPA Caused the Manifestation of Some Autistic-Like Behaviour in Young-Adult Offspring

**Figure 1:**
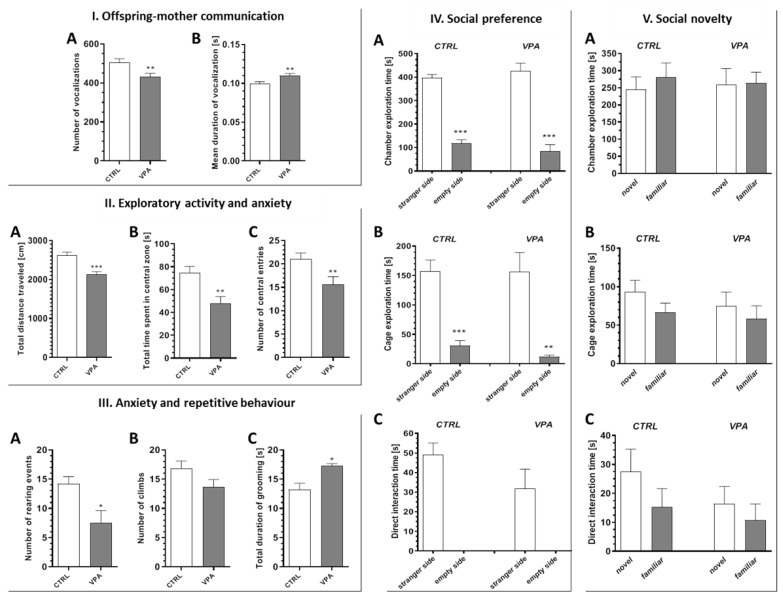
The effect of prenatal exposure to valproic acid (VPA) on offspring behaviour. (**I**). The impact of prenatal exposition to VPA on offspring–mother communication. The behaviour of control rats and rats exposed to VPA was analysed at postnatal day 11 in the juvenile isolation test. (A) The total number of ultrasonic vocalization events during the trial, *n* = 90 (CTRL), *n* = 88 (VPA); (B) the duration of average ultrasonic vocalization event, *n* = 90 (CTRL), *n* = 91 (VPA). (**II**). The impact of prenatal exposition to VPA on exploratory activity and anxiety. The behaviour of control rats and rats exposed to VPA was analysed at postnatal day 55 in the open-field test. (A) The total distance travelled by animals, *n* = 58 (CTRL), *n* = 48 (VPA); (B) the time spent in the central zone, *n* = 50 (CTRL), *n* = 39 (VPA); (C) the number of entries to the central zone, *n* = 45 (CTRL), *n* = 33 (VPA). (**III**). The impact of prenatal exposition to VPA on anxiety and repetitive behaviour. The behaviour of control rats and rats exposed to VPA was analysed at postnatal day 55 in the open-field test. (A) The number of rearing events, *n* = 10 (CTRL), *n* = 7 (VPA); (B) the number of climbing events, *n* = 10 (CTRL), *n* = 7 (VPA); (C) the total time spent on self-grooming, *n* = 9 (CTRL), *n* = 5 (VPA). Data represent the mean values ± SEM. (**IV**,**V**). The impact of prenatal exposition to VPA on social behaviour. The behaviour of control rats and rats exposed to VPA was analysed at postnatal day 57 in a three-chamber sociability and social novelty test (Crawley’s test). (**IV**). (A) The time spent on the exploration of each chamber during the social preference test, (B) the time spent on exploration of each cage during social preference test, and (C) the time spent on direct interaction with the other animal during social preference test. (**V**). (A) the time spent on the exploration of each chamber during a social novelty test, (B) the time spent on exploration of each cage during a social novelty test, and (C) the time spent on direct interaction with the other animal during a social novelty test. Data represent the mean values ± SEM from *n* = (5–8). * *p* < 0.05, ** *p* < 0.01, *** *p* < 0.001.

Animals prenatally exposed to VPA showed impairments in the two core symptoms related to ASD. The number of ultrasonic vocalisations (USV) emitted by juvenile rats separated from their mothers in the isolation test (ISO) was reduced (by about 14%, *p* = 0.0066) in the group subjected to VPA (Figure 1(IA)), whereas the duration of the vocalisation event was increased (by about 10%, *p* = 0.0074) (Figure 1(IB)), indicating autism-like impairment of the offspring–mother interaction. Additionally, in the open-field test (OF), rats subjected to VPA exhibited lower exploratory activity and increased anxiety. As shown on Figure 1(IIA), the total distance travelled by the rats prenatally subjected to VPA was shorter (by about 18%, *p* < 0.0001) compared to the control rats. Both the total time spent in the central zone and the number of entries to this zone was lower for the VPA-exposed rats (by about 36%, *p* = 0.0010 and by about 26%, *p* = 0.0089, respectively), suggesting an increased level of anxiety (Figure 1(IIB,IIC). The decreased rearing behaviour (by about 46%, *p* = 0.0109) and the tendency to decrease in the number of climbs are also indicative of increased anxiety, as is reduced exploration (Figure 1(IIIA,IIIB)). The total duration of self-grooming was increased (by about 30%, *p* = 0.0192) in VPA-treated rats, indicating an enhancement of their autism-like repetitive behaviour (Figure 1(IIIC)). In turn, the three-chamber sociability and social novelty test demonstrated that the rats subjected to VPA had no impairment in their social behaviour. In the social preference test (Figure 1(IVA–IVC)), both tested experimental groups spent significantly more time exploring the chamber containing the cage with a strange animal (social stimulus) compared to the chamber containing the empty cage (non-social stimulus) (*p* < 0.0001 in both groups); however, no detectable differences between groups were observed (Figure 1(IVA)). Moreover, the same results were obtained when the time spent on direct exploration of the cage (Figure 1(IVB)) (*p* < 0.0001 and *p* = 0.0023 in the control and VPA groups, respectively) or on direct interaction with the animal (Figure 1(IVC)) was analysed. In the social novelty test (Figure 1(VA–VC)), both the control and VPA rats spent the same amount of time exploring the chamber containing the cage with the novel animal (novel social stimulus) and with the known animal (familiar social stimulus) (Figure 1(VA)). The same results were obtained when the time spent on direct exploration of the cage (Figure 1(VB)) or on direct interaction with the animal was measured (Figure 1(VC)), but some weak tendencies were observed in both experimental groups for the novel animals.

### 2.2. Prenatal Exposure to VPAInduced Ultrastructural Changes in the Synapses of Young-Adult Offspring. The Features of Synaptic Pathology in TEM Analysis

**Figure 2:**
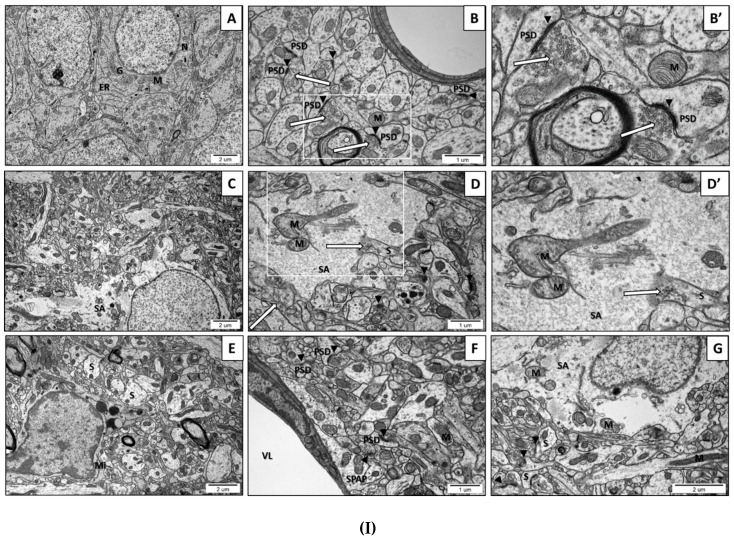

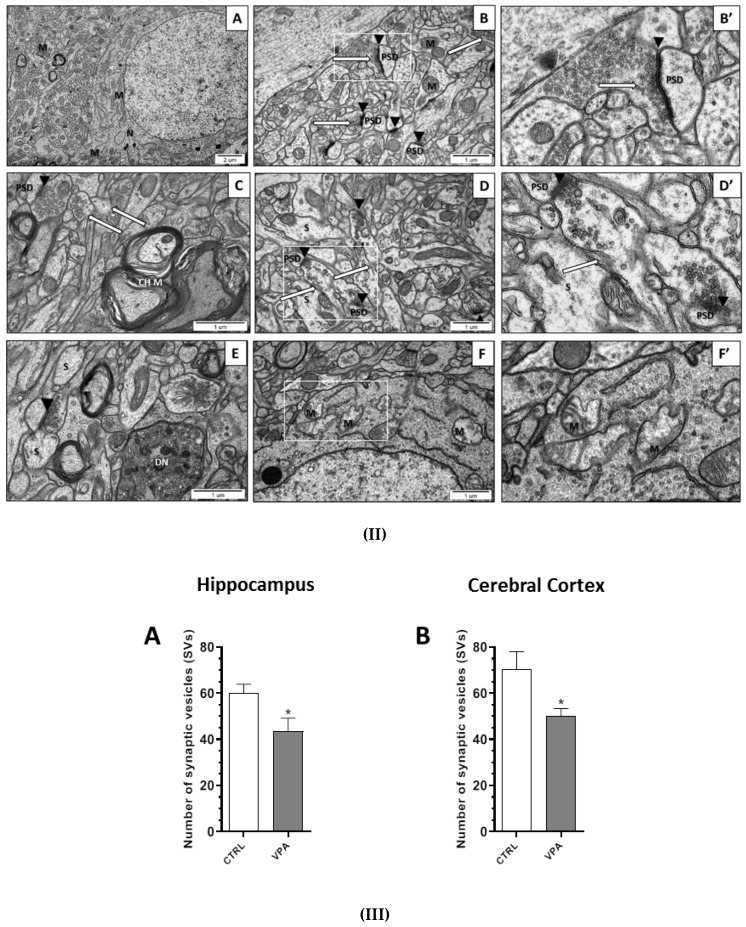
(**I**). The effect of prenatal exposure to VPA on the ultrastructure of neuronal cells in the CA1 region of the hippocampus of the offspring. (A–B’) Control group. Ultrastructurally unchanged neuronal cells, unaltered structure of neuropil with normal appearance of the synaptic cleft (black arrowheads), well-defined structure of the synapses with accurate postsynaptic density (PSD), the correct distribution of synaptic vesicles (SVs) (long arrows), and ultrastructurally unchanged mitochondria (M). (C–G) VPA-exposed group. Reduced packing density of SVs in the presynaptic area (release of SVs from the presynaptic area accompanied by disruption of the synaptic membranes) (long arrow), nerve ending swelling (S), blurred and thickened structure of the synaptic cleft without clearly marked pre- and postsynaptic membranes (black arrowheads). Ultrastructurally changed mitochondria with a blurred cristae structure (M). Astrocytes with features of swelling (SA) and swollen perivascular astrocyte processes (SPAP) were observed. Microglial cell activation (Mi); (VL) Vascular lumen; (G) Golgi apparatus; (ER) Endoplasmatic reticulum; (N) Neuron. Representative pictures from *n* = 6 independent experiments for the control and experimental animals are presented. (**II**). The effect of prenatal exposure to VPA on the ultrastructure of neuronal cells in the cerebral cortex of the offspring. (A–B’) Control group. Ultrastructurally unchanged neuronal cells, unaltered structure of neuropil with normal appearance of the synaptic cleft (black arrowheads), well-defined structure of synapses with accurate postsynaptic density (PSD), correct distribution of SVs (long arrows), and well preserved mitochondria (M). (C–F’) VPA-exposed group. Reduced packing density of SVs in the presynaptic area (release of SVs from the presynaptic area accompanied by the disruption of the synaptic membranes) (long arrows), nerve ending swelling (S), and blurred and thickened structure of the synaptic cleft, without clearly marked pre- and postsynaptic membranes (black arrowheads). Ultrastructurally changed mitochondria with a blurred cristae structure (M). Changed myelin structure (CHM); (DN) Degenerating neuron; (N) Neuron. Representative pictures from *n* = 6 independent experiments for the control and experimental animals are presented. (**III**). The effect of prenatal exposure to VPA on the synaptic vesicles (SVs) number. The effect of VPA on the number of synaptic vesicles (SVs) in the CA1 region of the hippocampus (A) and the cerebral cortex (B) was analysed at postnatal day 58 (PND58). The number of SVs was counted in 30 nerve endings of each animal from the control and experimental groups. Data represent the mean values ± SEM from *n* = 4 independent experiments. * *p* ˂ 0.05, vs. control.

The transmission electron microscopy (TEM) analysis of neurons and other brain cells in the CA1 region of the hippocampus and in the frontal cerebral cortex of the control rats indicated ultrastructurally unchanged neuropil with proper, well-defined structures of synapses with a correct distribution of the synaptic vesicles (SVs). Multiple vesicles were in direct contact with the presynaptic membrane. The synaptic cleft was narrow with prominent and clearly stained postsynaptic density (PSD). The nerve endings did not reveal the features of the swelling. The mitochondrial structure was unchanged (Figure 2(IA,IB,IB’) and Figure 2(IIA,IIB,IIB’)). However, the images of the brain parenchyma of rats prenatally exposed to VPA revealed some pathological changes in the ultrastructure of the synapses in all examined brain regions. In the brains of rats prenatally subjected to VPA we observed nerve ending swelling (S) and a blurred and thickened structure of the synaptic cleft, without clearly marked pre- and postsynaptic membranes (black arrowheads) (Figure 2(ID–IG) and Figure 2(IIC–IIE)). Furthermore, a greatly reduced packing density of SVs in the presynaptic area was visible (Figure 2(ID–IG) and Figure 2(IIC–IIE)). In some synapses of VPA rats, a complete lack of vesicles was observed in the presynaptic terminals. Additionally, in the vast majority of synapses, the release of SVs from the presynaptic area was accompanied by disruption of the synaptic membranes (long arrows) (Figure 2(ID’) and Figure 2(IIC,IID,IID’)). In order to statistically analyse the alterations in the amount of SVs in the synapses of VPA offspring, we randomly chose TEM images of synapses in the hippocampus and cerebral cortex neurons and calculated the total number of SVs in the presynaptic terminals. The quantitative analysis indicated a significant decrease in the number of SVs in the presynaptic area of both analysed structures in the offspring prenatally exposed to VPA (Figure 2(IIIA,IIIB)). Moreover, in the vast majority of synapses, the postsynaptic density (PSD) was blurred, appeared to be thickened, and possessed poorly discernible membranes (Figure 2(IF) and Figure 2(IID,IID’)). Furthermore, in both the cerebral cortex and hippocampus of VPA rats, we observed ultrastructurally altered mitochondria, some of which were elongated, swollen, or shrunken. The mitochondrial cristae and membrane were fused and blurred (M) (Figure 2(ID,ID’,IF,IG), and Figure 2(IIF,IIF’)). Moreover, exposure to VPA caused astrocyte swelling (SA) (Figure 2(IC,ID,IG)) and induced morphological changes in the structure of myelin (CHM) (Figure 2(IIC)).

### 2.3. Prenatal Exposure to VPA Altered the Levels of the Presynaptic Proteins: Synaptic Vesicle Proteins (v-SNARE) And Proteins Associated with The Presynaptic Membrane (t-SNARE)

**Figure 3:**
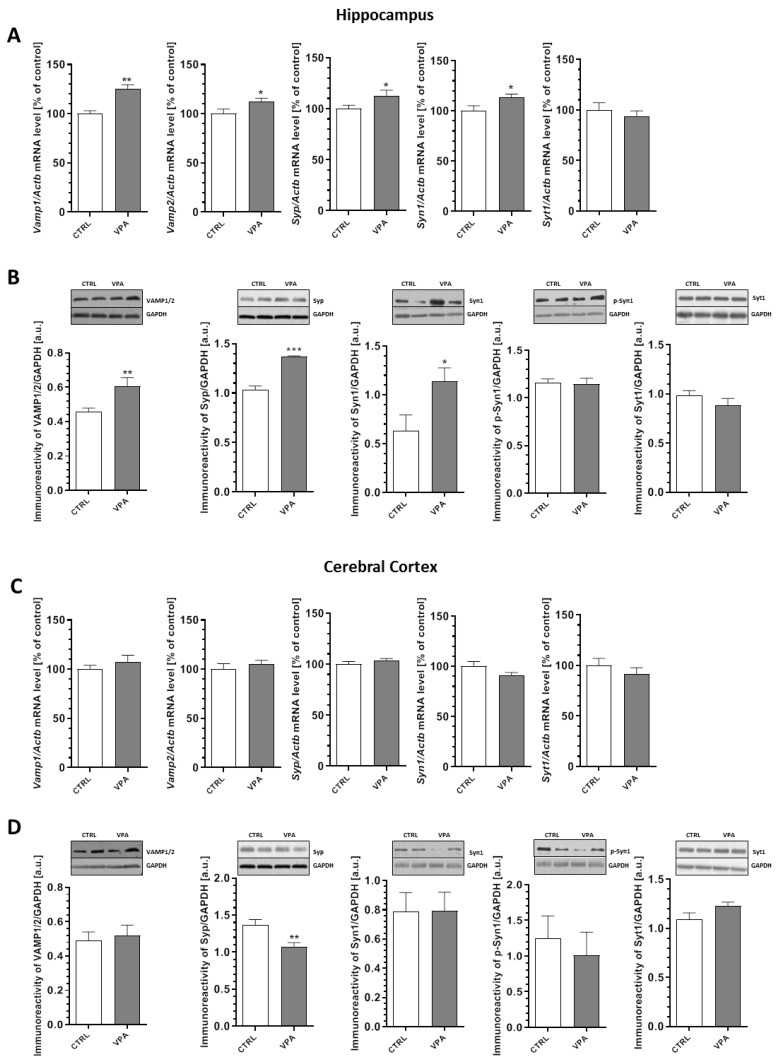
The effect of prenatal exposure to VPA on the expression of *v*-SNARE proteins: VAMP1/2, Syp, Syn1, p-Syn1(Ser62/Ser67), and Syt1. The level of mRNA of *Vamp1, Vamp2, Syp, Syn1,* and *Syt1* in the hippocampus (**A**) and cerebral cortex (**C**) of the control and VPA-exposed rats is presented. The level of mRNA was measured by real-time PCR and calculated by the ΔΔCt method with *Actb* (β-actin) as a reference gene. The data represent the mean values ± SEM from *n* = (4–5) independent experiments in the hippocampus and *n* = (4–6) in the cerebral cortex. The immunoreactivity of the *v*-SNARE proteins in the control and the VPA-exposed rats was monitored using a Western blot analysis. Densitometric analysis and representative pictures of VAMP1/2, Syp, Syn1, p-Syn1, and Syt1 in the hippocampus (**B**) and cerebral cortex (**D**) are shown. Results were normalized to GAPDH levels. Data represent the mean values ± SEM from *n* = (3–9) independent experiments in both the hippocampus and cerebral cortex. * *p* < 0.5, ** *p* < 0.01, *** *p* < 0.001 vs. control.

The changes in synaptic ultrastructure observed in the prenatally treated VPA rats encouraged us to investigate the possible effects of this compound at the molecular level. To evaluate if the morphological alterations of the synapses in the VPA offspring may be related to changes in the synaptic proteins, a quantitative analysis of the level of mRNA and a semi-quantitative analysis of the level of key presynaptic proteins were performed. We examined the expression of synaptic vesicle proteins (*v*-SNARE): synaptobrevin 1 and 2 (VAMP1/2), synaptophysin (Syp), synapsin 1 (Syn1), phospho-synapsin 1(Ser62/Ser67) (p-Syn1), and Ca^2+^-sensing synaptotagmin 1 (Syt1) together with the proteins associated with the presynaptic plasma membrane (*t*-SNARE): SNAP25 and syntaxin 1 (Stx1). Our data indicated a significant increase in both the gene expression and protein level of VAMP1/2 (an increase in *Vamp1* and *Vamp2* expression by about 25% (*p* = 0.0016) and 12% (*p* = 0.0290), respectively, together with a significant increase by 32% for the VAMP1/2 protein level (*p* = 0.0062)) in the hippocampus of the VPA offspring (Figure 3A,B). In addition, in the same brain structure we observed a significant (*p* < 0.001) increase of about 32% in the level of Syp with a concomitant rise by about 12% in the mRNA level (*p* = 0.0488) compared to the control group (Figure 3A,B). Moreover, exposure to VPA during foetal life induced elevation in both the *Syn1* gene expression (by about 14%, *p* = 0.0365) and the protein level of synapsin 1 (by about 80%, *p* = 0.0278) in the hippocampus (Figure 3A,B). The quantitative RT-PCR and Western blot analysis revealed a lack of changes in both mRNA and the protein level of p-Syn1 and Syt1 in the hippocampus of VPA-exposed animals (Figure 3A,B). On the other hand, in the cerebral cortex of VPA rats, we observed a significant (*p* = 0.0098) decrease by about 21% in the level of Syp without changes in the gene expression of this protein (Figure 3C,D). Moreover, prenatal exposure to VPA had no effect on the expression of VAMP1/2, Syn1, p-Syn1, and Syt1 in this brain structure (Figure 3C,D).

**Figure 4:**
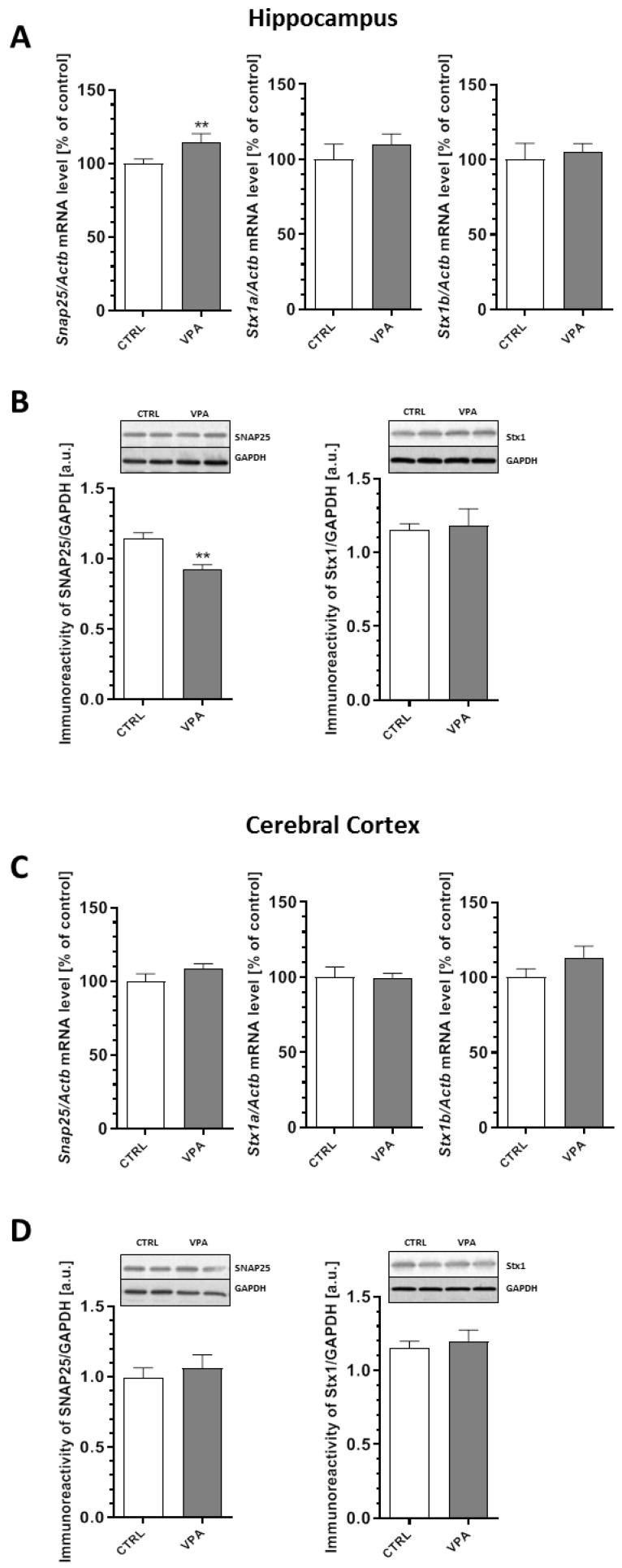
The effect of prenatal exposure to VPA on the expression of the *t*-SNARE proteins: SNAP25 and Stx1. The gene expression of *Snap25, Stx1a,* and *Stx1b* in the hippocampus (**A**) and cerebral cortex (**C**) of the control and VPA-exposed rats was measured via quantitative RT-PCR and calculated by the ΔΔCt method with *Actb* (β-actin) as a reference gene. Data represent the mean values ± SEM from *n* = (4–6) independent experiments in both the hippocampus and cerebral cortex. The immunoreactivity of the *t*-SNARE proteins in the control and the VPA-exposed rats was monitored using a Western blot analysis. Representative pictures and a densitometric analysis of SNAP25 and syntaxin 1 (Stx1) in the hippocampus (**B**) and cerebral cortex (**D**) are presented. The results were normalized to GAPDH levels. The data represent the mean values ± SEM from *n* = 5 independent experiments in both the hippocampus and cerebral cortex. ** *p* < 0.01, vs. control.

In turn, our analysis of expression of the presynaptic plasma membrane proteins revealed a significant (*p* = 0.0024) decrease of about 20% in the protein level of SNAP25 in the hippocampus of the VPA offspring despite the 15% increase (*p* = 0.0086) in *Snap25* gene expression (Figure 4A,B). Prenatal exposure to VPA had no effect on the expression of this protein in the cerebral cortex (Figure 4C,D). In addition, the quantitative RT-PCR and Western blot analysis revealed a lack of changes in *Stx1a* and *Stx1b* mRNA expression, as well as the protein level of Stx1 in both examined brain structures of the young-adult offspring prenatally exposed to VPA (Figure 4A–D).

### 2.4. Prenatal Exposure to VPA Evoked Changes in the Level of Postsynaptic Density (PSD) Proteins and Synaptic Cell Adhesion Molecules

**Figure 5:**
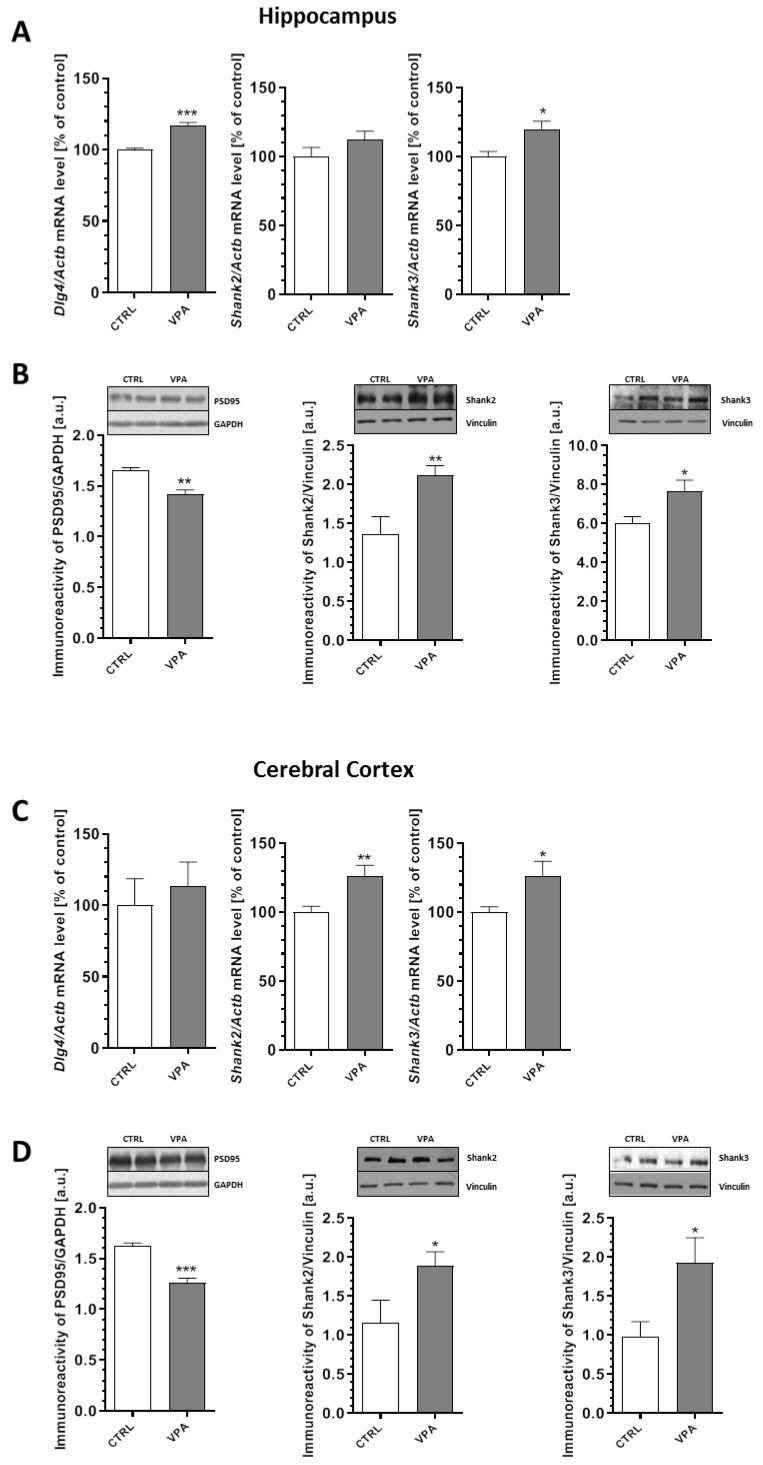
The effect of prenatal exposure to VPA on the expression of the postsynaptic density proteins: PSD95, Shank2, and Shank3. The gene expression of *Dlg4, Shank2,* and *Shank3* in the hippocampus (**A**) and cerebral cortex (**C**) of the control and VPA-exposed rats was measured by quantitative RT-PCR and calculated by the ΔΔCt method with *Actb* (β-actin) as a reference gene. Data represent the mean values ± SEM from *n* = (4–5) independent experiments in the hippocampus and *n* = (4–8) in cortex. The immunoreactivity of the postsynaptic density proteins in the control and VPA-exposed rats was monitored using Western blot analysis. Densitometric analysis and representative pictures for PSD95, Shank2, and Shank3 in the hippocampus (**B**) and cerebral cortex (**D**) are presented. The results were normalized to GAPDH or vinculin levels. Data represent the mean values ± SEM from *n* = (4–9) independent experiments in both the hippocampus and cerebral cortex. * *p* < 0.05, ** *p* < 0.01, *** *p* < 0.001 vs. control.

In the next step, we examined the expression of the crucial postsynaptic scaffolding proteins that are essential for efficient neurotransmission, synaptogenesis, and neurodevelopment: postsynaptic density protein 95 (PSD95) and the SH3 domain and ankyrin repeat-containing protein (Shank). The effect of prenatal exposure to VPA on the expression and protein level of PSD95, Shank2, and Shank3 in the hippocampus and cerebral cortex are summarized in Figure 5. Our data indicated that exposure to VPA during embryonic development significantly reduced the level of PSD95 by about 14% in the hippocampus (*p* = 0.0024) and by about 22% in the cerebral cortex (*p* = 0.0003) (Figure 5B,D). A decreased level of PSD95 in the hippocampus was accompanied by an increase of its gene expression by about 17% (*p* = 0.0003) (Figure 5A). In turn, in the cerebral cortex, *Dlg4* expression was unchanged compared to the control group (Figure 5C). Moreover, prenatal exposure to VPA evoked a significant increase in the level of both the Shank2 and Shank3 proteins in the hippocampus (a 56% increase in Shank2, *p* = 0.0028 and 27% in Shank3, *p* = 0.0161) and cerebral cortex (63% increase in Shank2, *p* = 0.0248 and 96% in Shank3, *p* = 0.0151) compared to the respective control groups (Figure 5B,D). An increase in the protein level of Shank2 and Shank3 in the cerebral cortex was linked with an increase in both the *Shank2* (*p* = 0.0048) and *Shank3* (*p* = 0.0345) mRNA levels (Figure 5C). In the hippocampus, an increase in the protein level of Shank3 was accompanied by an elevation of its gene expression by 20% (*p* = 0.0143) (Figure 5A), but *Shank2* gene expression remained unchanged (Figure 5A).

**Figure 6:**
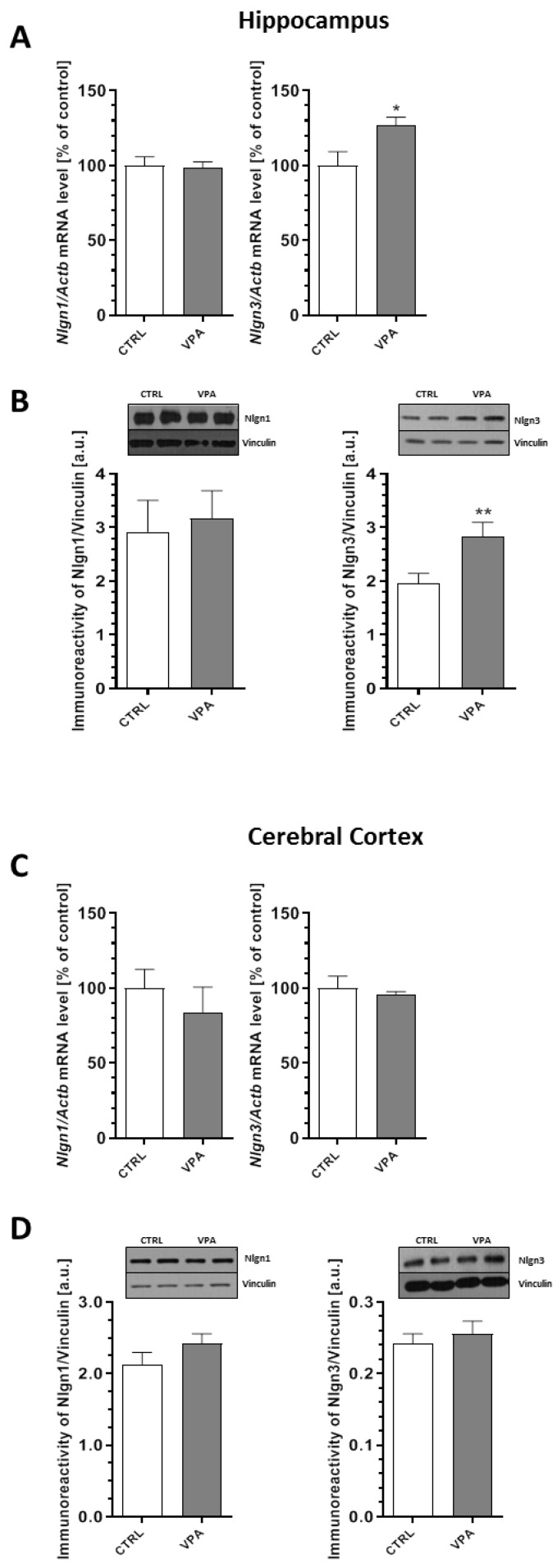
The effect of prenatal exposure to VPA on the expression of the synaptic cell adhesion molecules: Nlgn1 and Nlgn3. The gene expression of *Nlgn1* and *Nlgn3* in the hippocampus (**A**) and cerebral cortex (**C**) of the control and VPA-exposed rats was measured with quantitative RT-PCR and calculated by the ΔΔCt method with *Actb* (β-actin) as a reference gene. The data represent the mean values ± SEM from *n* = (5–6) independent experiments in both the hippocampus and cerebral cortex. The immunoreactivity of Nlgn1 and Nlgn3 in the control and VPA-exposed brains was monitored using a Western blot analysis. Densitometric analysis and representative pictures for Nlgn1 and Nlgn3 in the hippocampus (**B**) and cerebral cortex (**D**) are shown. Results were normalized to vinculin levels. Data represent the mean values ± SEM from *n* = (11–14) independent experiments in both the hippocampus and cerebral cortex. * *p* < 0.5, ** *p* < 0.01 vs. control.

Additionally, we performed qRT-PCR and Western blot analysis of the synaptic cell adhesion molecules known as neuroligins (Nlgns), which are responsible for the connection between two neurons and the formation, maturation, and stabilization of synapses. In our study, the qRT-PCR and Western blot analysis revealed the lack of changes in both the mRNA and protein levels of Nlgn1 in the hippocampus and cerebral cortex of offspring prenatally exposed to VPA (Figure 6A–D). In turn, prenatal exposure to VPA significantly increased the level of neuroligin3 (Nlgn3) by about 45% in the hippocampus (*p* = 0.0079) (Figure 6B), which was accompanied by an increase in its gene expression (*Nlgn3*) of about 27% (*p* = 0.0165) (Figure 6A). Analysis of the expression of Nlgn3 in the cerebral cortex revealed the lack of changes in both the mRNA and protein levels in animals exposed to VPA during embryonic development (Figure 6C,D).

### 2.5. Prenatal Exposure to VPA Evoked Oxidative Stress and ROS Generation in the Brains of Young-Adult Offspring

**Figure 7:**
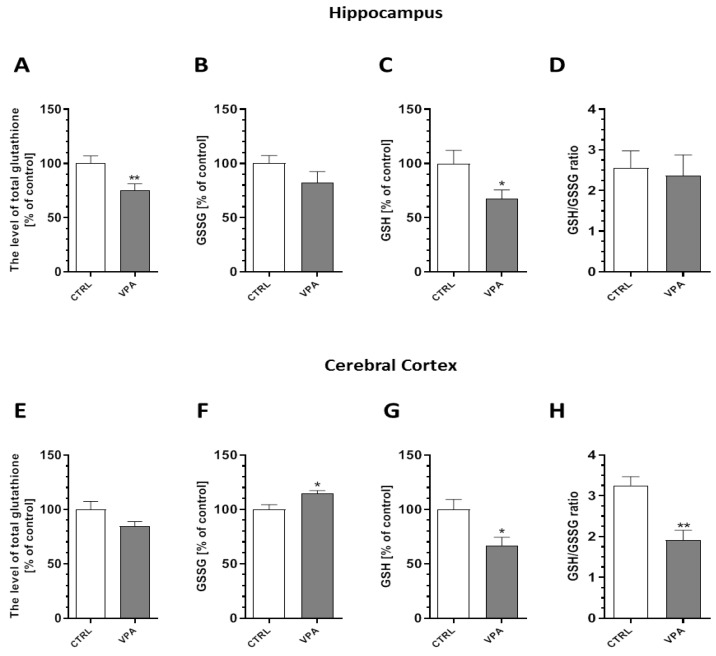
The effect of prenatal exposure to VPA on the glutathione level. The glutathione redox imbalance was determined by spectrophotometric assays. We determined the level of total glutathione, oxidized glutathione (GSSG), reduced glutathione (GSH), and the ratio of GSH/GSSG in the hippocampus ((**A**–**D**), respectively), as well as in the cerebral cortex ((**E**–**H**), respectively), of the control and VPA-exposed animals. Data represent the mean values ± SEM from *n* = (10–12) independent experiments in the hippocampus and *n* = (3–5) in the cerebral cortex. * *p* < 0.05, ** *p* < 0.01 vs. control.

**Figure 8:**
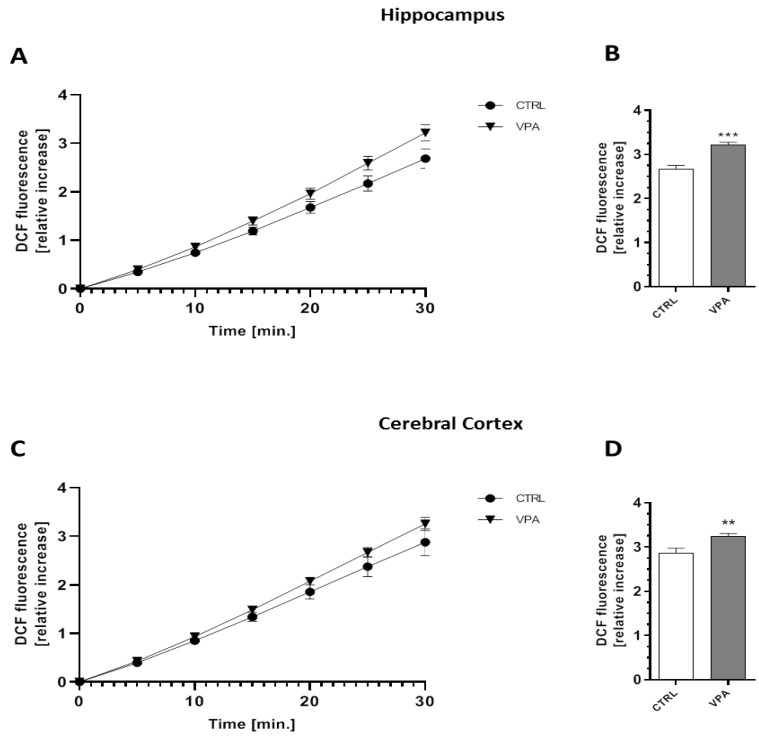
The effect of prenatal exposure to VPA on free oxygen radicals (ROS) generation. The level of ROS was determined using a fluorescent probe (H_2_DCF-DA) on the hippocampus and cerebral cortex of the control and VPA-exposed animals for 30 min ((**A**,**C**), respectively) and after 30 min ((**B**,**D**), respectively). The results are presented as the relative increase in fluorescence. Data represent the mean values ± SEM from *n* = 8 independent experiments in both the hippocampus and cerebral cortex. ** *p* < 0.01, *** *p* < 0.001 vs. control.

To evaluate whether prenatal VPA exposure leads to the induction of oxidative stress, we measured the total glutathione (GSH) and oxidized glutathione (GSSG) content in both the hippocampus and the cerebral cortex of young-adult rats. As shown in Figure 7, the level of total GSH, the major antioxidant in the brain, was markedly reduced by about 25% (*p* = 0.0072) in the hippocampus (Figure 7A). In the same brain structure, we also detected a significant (*p* = 0.0226) decrease in the level of reduced GSH of about 32%, whereas both the level of GSSG and the ratio of GSH/GSSG remained unchanged (Figure 7B–D). In turn, in the cerebral cortex of VPA offspring, despite the lack of changes in the total level of GSH, the level of GSSG was significantly (*p* = 0.0242) increased by about 15% compared to the offspring of the control dams (Figure 7E,F). Moreover, the level of reduced glutathione and the ratio of GSH/GSSG were markedly decreased (by about 33%, *p* = 0.0221 and by about 41%, *p* = 0.0045, respectively) (Figure 7G,H). To confirm the pro-oxidative properties of VPA, its effect on the level of free oxygen radicals (ROS) was investigated using a fluorescent probe H_2_DCF-DA in an ex vivo system. As shown in Figure 8, prenatal exposure to VPA evoked the generation of ROS (a significant increase in the fluorescence of the DCF probe) in both hippocampus by about 20% (*p* = 0.0001) (Figure 8A,B) and by about 14% (*p* = 0.0037) in cerebral cortex (Figure 8C,D) of young-adult offspring compared to the control.

### 2.6. Prenatal Exposure to VPA Affected the Microglia in the Young-Adult Offspring

**Figure 9:**
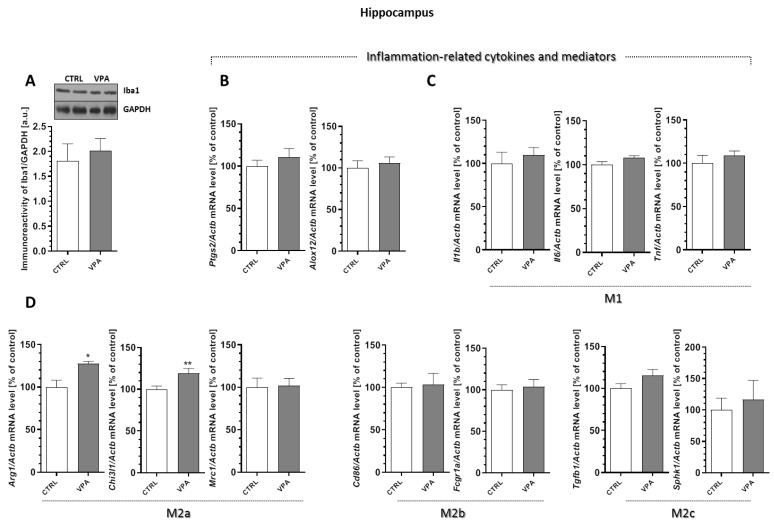
The effect of prenatal exposure to VPA on the status of microglia in the hippocampus. (**A**) The immunoreactivity of Iba-1 was monitored using a Western blot analysis. Densitometric analysis and representative pictures are shown. Results were normalized to GAPDH levels. Data represent the mean values ± SEM from *n* = (6–8). independent experiments. The gene expression of *Ptgs2*, *Alox12,* (**B**); *Il1b, Il6,* and *Tnf* (**C**); and the markers of the M2 phenotype’s microglia activation: *Arg1, Chi3l1, Mrc1, Cd86, Fcgr1a, Tgfb1,* and *Sphk1* (**D**), in the hippocampus of the control and VPA-exposed rats were measured with quantitative RT-PCR and calculated by the ΔΔCt method using *Actb* (β-actin) as a reference gene. Data represent the mean values ± SEM from *n* = (4–6) independent experiments. * *p* < 0.5, ** *p* < 0.01 vs. control.

**Figure 10:**
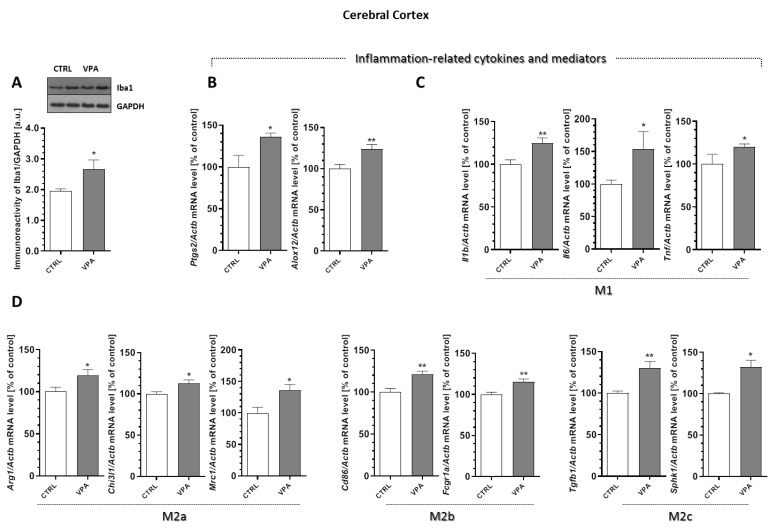
The effect of prenatal exposure to VPA on the status of microglia in the cerebral cortex. (**A**) The immunoreactivity of Iba-1 was monitored using a Western blot analysis. Densitometric analysis and representative pictures are shown. Results were normalized to GAPDH levels. Data represent the mean values ± SEM from *n* = (8–11) independent experiments. The gene expression of *Ptgs2, Alox12* (**B**); *Il1b, Il6,* and *Tnf* (**C**); and the markers of the M2 phenotype’s microglia activation: *Arg1, Chi3l1, Mrc1, Cd86, Fcgr1α, Tgfb1,* and *Sphk1* (**D**) in the cerebral cortex of the control and VPA-exposed rats were measured with quantitative RT-PCR and calculated by the ΔΔCt method with *Actb* (β-actin) as a reference gene. Data represent the mean values ± SEM for *n* = (3–6) independent experiments. * *p* < 0.5, ** *p* < 0.01 vs. control.

To analyse the microglia’s response to the changes induced by VPA, the protein level of Iba-1, a Ca^2+^-binding protein constitutively expressed by both surveillant and activated microglia was determined. Moreover, for additional evaluations of the status of microglia cells, the mRNA levels of the selected pro-inflammatory cytokines (the markers of cytotoxic microglia activation phenotype M1 (*Il1b, Il6,* and *Tnf*) and the markers of the cytoprotective M2 phenotype (*Arg1, Chi3l1, Mrc1, Cd86, Fcgr1a, Tgfb1,* and *Sphk1*)) were analysed. Our results indicated a lack of changes in the protein level of Iba-1 in the hippocampus of the VPA offspring (Figure 9A). Furthermore, the gene expression of the enzymes that are responsible for the production of the mediators of inflammation, cyclooxygenase-2 (*Ptgs2*) and 12-lipoxygenase (*Alox12*), as well as the pro-inflammatory cytokines *Il1b, Il6,* and *Tnf*, remained unchanged in this brain structure in response to prenatal VPA exposure (Figure 9B,C). In turn, the analysis of the markers of the M2 phenotype revealed a significant increase in the mRNA levels of *Arg1* (by about 27%, *p* = 0.0190) and *Chi3l1* (by about 19%, *p* = 0.0082) in the hippocampus of VPA offspring (Figure 9D). A completely different response of the microglia was observed in the cerebral cortex of the VPA offspring. In this part of the brain, we noted a significant (*p* = 0.0276) increase in the protein level of Iba-1 of about 37% compared to the control (Figure 10A). VPA also affected the expression of cyclooxygenase-2 (COX-2) and 12-lipoxygenase (12-LOX). Compared to the control groups, the expression of *Ptgs2* and *Alox12* was significantly increased in the cerebral cortex by about 36% (*p* = 0.0192) and 24% (*p* = 0.0059), respectively (Figure 10B). Together with increased expression of the enzymes responsible for the production of the mediators of inflammation, we observed a significant increase in the mRNA level of pro-inflammatory cytokines *Il1b* (about 25%, *p* = 0.0022), *Il6* (about 54%, *p* = 0.0275), and *Tnf* (about 19%, *p* = 0.0406) (Figure 10C), suggesting activation of the local inflammatory response in this brain structure. Interestingly, in this part of the brain, prenatal exposure to VPA also stimulated the gene expression of all analysed markers of alternative, anti-inflammatory, and neuroprotective M2 phenotypes of microglia (M2a, M2b, and M2c subtypes) (Figure 10D). The gene expression of *Arg1, Chi3l1, Mrc1, Cd86, Fcgr1a, Tgfb1,* and *Sphk1* was significantly elevated (by about 19%, 12%, 36%, 21%, 15%, 31%, and 32%, respectively) compared to the control (Figure 10D).

## 3. Discussion

In this study, we used an environmentally triggered rat model of ASD based on prenatal exposure to valproic acid (VPA) [31]. In humans, VPA is used to treat bipolar disorder and epilepsy and also to prevent migraine headaches. However, using medication with VPA during early pregnancy increases the risk of giving birth to a child with ASD. The rat VPA-based model reproduces several pathological, anatomical, and behavioural features of ASD.

To characterize our model of environmentally-triggered autism, we performed a panel of behavioural tests to evaluate crucial ASD-related deficits. Our results demonstrated some behavioural alterations both in juvenile and young-adult VPA-exposed animals. A juvenile isolation test was used to analyse communication deficits between animals [32]. In accordance with other data [4,33], the results demonstrated impairment of offspring–mother communication, which resembles typical ASD developmental social communication deficits. In the open-field test, we observed a reduction in exploratory activity and increased anxiety in the animals prenatally exposed to VPA, which is also comparable to other published data [31,34]. However, in our experimental model, we did not observe any sign of deficits in social behaviour. In the present study, we used an intraperitoneal dose of 450 mg/kg of VPA because higher doses lead to foetal resorption [35]. In some studies, this dose evoked a behavioural deficit [34]. However, in other studies, VPA had no effect and even increased social behaviour. It seems that the effect of prenatal VPA exposure on social behaviour in rodents is strictly dependent on the dose of VPA. Prenatal exposure to high doses (500–600 mg/kg) of VPA decreases social interaction, whereas lower doses tend to increase social behaviour [31,36,37,38,39]. Interestingly, some studies reported increased sociability after a high dose of VPA (600 mg/kg [40]). Even if the precise mechanism of this dose-dependent effect of VPA on behaviour is not fully understood, it is likely that dose-dependent alterations of synaptic proteins may affect synaptic development in diverse brain circuits.

This study shows the significant pathology of the synaptic ultrastructure and variations in the expression of key synaptic proteins in the brains of young-adult rat offspring prenatally exposed to VPA as an environmentally induced rodent model of autism. This model better represents many features of idiopathic autism of environmental/epigenetic origin than transgenic models based on mutations in single autism-associated genes. A prenatal environmental insult changes the structure of synapses in a mature brain, such as producing swollen synapses with a significantly reduced packing density of SVs and a blurred and thickened structure of the synaptic cleft, without clearly marked pre- and postsynaptic membranes. Moreover, in most synapses, the postsynaptic density is blurred and appears to be thickened and possesses hardly discernible membrane. Furthermore, a TEM study showed structural alterations in the synaptic mitochondria, which were shrunken, swollen, or elongated. All these observations clearly indicate that synaptic organization is changed in young-adult offspring prenatally exposed to VPA. These structural abnormalities were accompanied by disturbances of key pre- and postsynaptic proteins, including VAMP1/2, Syp, Syn1, SNAP25, PSD95, Shank2, Shank3, and Nlgn3, in the hippocampus, as well as Syp, PSD95, Shank2, and Shank3 in the cerebral cortex.

All these synaptic abnormalities indicate that, under conditions of prenatal exposure to VPA, damage to synaptic function may occur, resulting in the impairment of synaptic plasticity and neurotransmission. Synaptic dysfunction, which ultimately leads to functional and cognitive impairments, is a fundamental causative factor and key mechanism underlying ASD pathology and many other neuropsychiatric disorders and cognitive dysfunctions [41]. Importantly, human studies revealed the exquisite sensitivity of cognitive function to precise levels of many diverse synaptic proteins [1].

The synaptic transmission underlying learning and memory formation requires the formation of membrane fusion machinery (SNARE complex) and the combined action between the presynaptic proteins and the proteins forming postsynaptic densities [30]. Therefore, any changes in the structure, function, or level of these proteins may lead to damage of the synaptic vesicle exocytosis, and neurotransmitter release [20].

Excessive expression of SV-associated proteins (VAMP1/2, Syp, and Syn1) together with a significant reduction of the protein level of SNAP25 in the hippocampus of VPA offspring could lead to disturbances in the docking and fusion of SVs and thereby synaptic exocytosis. However, a decrease in the protein level of SNAP25 was not correlated with changes in gene expression. The reason for this phenomenon could be various post-transcriptional or post-translational modifications, as well as the intensified or inhibited process of protein degradation. SNAP25, the protein associated with the presynaptic plasma membrane, which is necessary for SNARE complex formation, plays a crucial role in the CNS, mediating the docking of SVs in the presynaptic neurons, and contributing to the exocytosis that is essential for neurotransmitter release [3,20]. Moreover, SNAP25 seems to play an important role in synaptogenesis and is necessary for cognitive functioning and long-term memory consolidation [30]. Therefore, it is possible that the down-regulation of SNAP25 in the hippocampus of VPA offspring could contribute to presynaptic membrane dysfunction and may explain the pathological alterations in the structure of the synaptic cleft (blurred and not visible). Disruption of the presynaptic plasma membrane function associated with deficiency in the protein level of SNAP25 may also explain the significant decrease in the number of SVs observed in the presynaptic area of the hippocampal synapses. Despite the noted increase in the level of SV-associated proteins, we observed a reduced density of SVs in the presynaptic terminals of VPA rats. It is possible that a decrease in the amount of SVs may be associated with disruption of the presynaptic plasma membrane and “leakage” of SVs. Pathological alterations in the structure of the synaptic cleft, disruption of the synaptic membrane, and a decrease in the amount of SVs in the presynaptic area were also observed in the cerebral cortex of the offspring prenatally exposed to VPA. However, the lack of changes in the expression of the proteins associated with the presynaptic membrane (exclusively decreased level of Syp) indicates the involvement of other proteins and/or different molecular mechanisms underlying synaptic pathology in this part of the brain. Presynaptic synaptophysin, known as a major marker of SVs, forms a multimeric complex with VAMP1/2 in a 1:2 stoichiometric ratio. A decrease in the level of Syp together with the lack of changes in VAMP1/2 expression noted in the cortical synapses may suggest that the balance between Syp/VAMP1/2 is disturbed. Even small alterations in the Syp level may affect the targeting of VAMP1/2 to SVs and, consequently, may result in decreased fidelity of neurotransmission and synaptic dysfunction [42]. Although the genes encoding the presynaptic proteins have been studied in relation to different neurodevelopmental/psychiatric disorders, including ASD, the expression of the key proteins responsible for synapse formation, maintenance of their proper structure, and neurotransmitter release was not investigated. So far, the single nucleotide polymorphisms and mutations in the genes coding synaptic proteins have been examined [3,13,20,21,22,23,43,44,45,46]. This suggests that the synapse is a possible site of autism origin.

The process of synaptic transmission requires the proper interaction between proteins of the pre- and postsynaptic terminals. One of the most abundant proteins found in the postsynaptic density of excitatory synapses is PSD95 [13,20,47]. This scaffolding protein has been implicated in the regulation of long-term neuronal synaptic plasticity, which is associated with NMDA and AMPA receptor signalling [48,49] and dendritic spine morphogenesis during neurodevelopment and also serves as a major functional bridge interconnecting the neurexin-neuroligin-Shank pathway, thus promoting synapse stability [21,47]. Therefore, it is plausible that PSD95 dysfunction during development may alter synaptic plasticity events at the dendritic spines that contribute to the malformations of the synapses associated with neurological disorders. A reduction of PSD95 has been observed in many pathological conditions of the brain, including Alzheimer’s disease (AD), Parkinson’s disease (PD), schizophrenia, fragile X syndrome (FXS), and intellectual disability [50,51,52,53,54,55,56]. The dysregulation of PSD95 and Shank proteins observed in our study may suggest alterations of excitatory neurotransmission in VPA offspring. There is increasing evidence from human and animal studies suggesting that PSD95 disruption is linked to the pathologies of schizophrenia and autism [18]. PSD95 deletion (*Dlg4*^−/−^) in mice has been shown to exhibit multiple behavioural and molecular abnormalities relevant to ASD. Increased repetitive behaviours, abnormal communication and social interactions, impaired motor coordination, increased stress reactivity, and anxiety-related responses were shown in *Dlg4* knockout mice [57]. Every change in the level of synaptic PSD95 may affect the interactions with its partners and contribute to the development of CNS diseases. PSD95 has been also shown to be involved in a network of interactions with high-risk ASD genes that include Shank family genes and neuroligins [58].

Shank proteins are expressed in areas of the brain that are essential for cognition and learning and play a crucial role in spine formation and maturation, synaptogenesis, and neurodevelopment. Numerous lines of evidence suggest a strong relationship between mutations in *SHANK* family genes and the development of syndromic and idiopathic ASD [19,59,60,61]. Studies in vitro and in vivo highlight the important role of Shank3 in synaptic function [1]. Mutation in the *SHANK3* human gene leads to different neuropsychiatric diseases, including ASD, schizophrenia, intellectual disability, and Phelan–McDermid syndrome [13,59]. Evidence of the involvement of this protein in speech and intellectual deficits comes from the chromosomal translocation in 22q13.3 (ASD-linked region) that disrupts the *SHANK3* gene [41]. The loss of *Shank3* in the cultured hippocampal neurons of rats results in a decrease in both the length and density of spines. Conversely, the overexpression of *Shank3* in vitro promotes the maturation and enlargement of dendritic spines [62]. Deletion of the *Shank3* gene in mice leads to an abnormal postsynaptic structure and dysfunction of synaptic transmission, behaviour, and development [17,63]. In addition, deletions and a stop mutation in the *SHANK2* gene were also found in ASD patients. *Shank2* null mice presented fewer dendritic spines and reduced basal synaptic transmission, reduced NMDA receptor function, and abnormalities in their vocal and social behaviour [20]. All these studies fully establish that deregulation of Shank may underlie the aetiology of ASD. Shank controls multiple parameters of synapse biology in a dose-dependent manner. In the first animal model (lacking all Shank proteins and using the *Drosophila* neuromuscular junction (NMJ)) as a model of a glutamatergic synapse, the authors showed that both a lack of Shank proteins and their excessive expression is critical to synaptic development. A deficiency in Shanks impairs the number and maturity of synaptic boutons (axon terminals); in turn, their overexpression has morphological consequences similar to the loss of Shank [64]. In our study, we observed a significant increase in the protein level of Shank2 and Shank3 in the hippocampus of VPA offspring, among which only the increase in the Shank3 protein level was associated with a concomitant rise in mRNA expression. In turn, in the cerebral cortex of VPA-affected offspring, the level of both Shank2 and Shank3 was elevated with a simultaneous increase in gene expression. Some findings indicate that Shank3 overexpression causes manic-like behaviour and seizures consistent with a synaptic excitatory/inhibitory (E/I) imbalance, a key mechanism implicated in ASD [65]. Interestingly, the study of Arons et al. 2012 indicated that Shank3 levels may influence various facets of presynaptic function, perhaps through the activation of trans-synaptic neurexin/neuroligin complexes. These authors revealed coordinated increases in a variety of presynaptic proteins (VGLUT1, synaptophysin, as well as synapsin and VAMP2) in the synapses overexpressing EGFP-ProSAP2 [66], just as we observed in our study.

Neuroligins (Nlgns) are synaptic cell adhesion molecules enriched in the postsynaptic membranes that interact with neurexins. Neuroligin–neurexin interactions regulate various aspects of both excitatory (E) and inhibitory (I) synaptic development and function, affecting the excitatory–inhibitory (E/I) balance [67]. Recent studies have identified many mutations in the genes encoding neuroligins (e.g., R451C for *NLGN3* and a frame shift insertion mutation for *NLGN4*) linked to patients with ASD, intellectual disability, and schizophrenia [10,11,12,24,68]. Our study showed a significant increase in the protein level of neuroligin3 that was associated with excessive *Nlgn3* expression in the hippocampus of young-adult offspring rats subjected prenatally to VPA. However, Kolozsi et al. 2009 showed the significantly reduced mRNA expression of *Nlgn3* in all hippocampal regions of adult mice prenatally exposed to VPA [69]. The divergences in the results may be due to differences in the experimental model of VPA toxicity (period of exposure, species, and dose). Moreover, in the above-mentioned study of Kolozsi et al., the protein level of Nlgn3 was not examined.

In addition to synaptic dysfunction (a key mechanism in the pathophysiology of ASD), several studies have suggested that oxidative stress and neuroinflammation are important contributors to the pathology of ASD [70]. Our study revealed that ROS generation in both the hippocampus and cerebral cortex was significantly increased in animals prenatally exposed to VPA. Oxidative stress may be the basis for explaining the pathological changes in synaptic endings observed in our study. Altered GSH concentrations were demonstrated in the *post-mortem* brains of ASD patients [71]. Moreover, there is evidence of lower concentrations of GSH, higher levels of GSSG, and a decrease in the GSH/GSSG ratio in individuals with ASD [71,72,73]. The association between increased oxidative stress and decreased GSH levels has also been postulated to contribute to the pathogenesis of many neurological diseases, such as AD, PD, amyotrophic lateral sclerosis (ALS), multiple sclerosis, depression, and memory loss [74].

Glutathione is imperative for the regulation, response, and maintenance of the immune system; it also modulates the effect of inflammatory cytokines. Low GSH levels may underlie many of the systemic abnormalities associated with ASD, such as immune dysfunction/inflammation (impaired or altered immune response and the deregulation of pro-inflammatory cytokines) [75]. Chronic oxidative stress could be a consequence but also a cause of neuroinflammation. At the molecular level, oxidative stress induces inflammation via the activation of NF-κB, a transcriptional activator of the inflammatory response that can also induce the production of more free radicals [76,77,78]. Among the many inflammatory factors found during the process of “neuroinflammation”, inducible COX-2 is believed to be a critical enzyme expressed in response to cytokines and pro-inflammatory molecules [79]. Our results demonstrated the upregulation of *Ptgs2* and *Alox12* in the cerebral cortex of offspring prenatally exposed to VPA, supporting the role of both enzymes in VPA-evoked oxidative stress-mediated neuroinflammation.

Even low-level inflammation in the brain is detrimental for synaptic function and could lead to cognitive dysfunction and behavioural abnormalities [80]. The immune system in the CNS is represented by microglial cells, which constitute the first line of defence against pathological changes [4,81,82]. In postnatal life, microglia play a crucial role in sensing perturbations in the encephalic environment, actively responding to even minor pathological changes in the CNS by altering their shape and gene expression profile [6]. Under stress, microglial cells are induced into the M1 phenotype, releasing inflammatory mediators (IL-1β, IL-6, TNF-α, IFN-γ, CD68, and nitric oxide) and causing neuroinflammatory responses. After the inflammation fades away, microglia shift into the alternative activated M2 profile. This phenotype, considered anti-inflammatory and neuroprotective (which is typical for microglia cells expressing, for example, arginase-1 (ARG1), chitinase-like 3 proteins (CHI3L1), mannose receptor C-type 1 (MRC1), transforming growth factor β (TGF-β), interleukin 4 (IL-4), and interleukin 10 (IL-10)), is important for antagonizing inflammatory-induced damages in the CNS [81,83,84]. Changes in the microglia profile are correlated with the type of challenge faced by the CNS [6]. To detail, the exploration of phenotype polarization of microglial activation in our experimental conditions, an analysis of the genetic markers of M1 and M2 was performed. Moreover, a marker that stains both resting and activated microglia, Iba-1 was evaluated [85]. In our experiments we observed a significant increase in the protein level of Iba-1 in the cerebral cortex of VPA offspring, which could reflect a change in the number of microglial cells (their mobilization). In turn, the analysis of the gene expression of pro-inflammatory cytokines revealed a significant increase in the mRNA of *Il1b, Il6,* and *Tnf* in this brain structure. This suggests an activation of local microglia-associated inflammation in response to or in addition to the oxidative stress in the cerebral cortex of VPA offspring during foetal life. Similar changes were reported in ASD subjects. High levels of various pro-inflammatory cytokines, such as IL-1β, IL-6, and TNF-α have been reported in the *post-mortem* brain tissue and blood of autistic subjects [70,86,87]. In the VPA animal model, the levels of IL-1β, IL-6, and TNF-α were increased in the hippocampus [88] in a whole-brain homogenate [89]. Increased levels of IL-1β and IL-6 are associated with increased stereotypy, impaired cognitive abilities, anxiety, and decreased social interactions [6]. Interestingly, together with changes in pro-inflammatory cytokine expression, prenatal exposure to VPA also altered the expression of key markers of alternative, anti-inflammatory, and neuroprotective microglia (M2 phenotype) in the hippocampus and in the cortex. The gene expression of all analysed markers of the M2 phenotype (M2a, M2b, and M2c subtypes): *Arg1, Chi3l1, Mrc1, Cd86, Fcgr1a, Tgfb1,* and *Sphk1,* was elevated in the cerebral cortex of the offspring prenatally exposed to VPA, whereas in the hippocampus, only upregulation of the *Arg1* and *Chi3l1* gene was observed. Thus, in the cerebral cortex, the anti-inflammatory and compensatory response from the microglial cells seems to be stronger. This is possibly a response caused by the wider scope of the pathological changes observed in the cerebral cortex than in the hippocampus. All these data indicated that prenatal exposure to VPA induced long-lasting changes in the microglia status in a brain structure-specific manner. In the cerebral cortex, both a pro-inflammatory and a potentially beneficial recovery-promoting microglia phenotype were activated, while in the hippocampus, only an M2 phenotype was stimulated. To restore homeostasis in the brain, the microglia phenotype in the cerebral cortex likely switched from pro-inflammatory M1 to neuroprotective M2 during the pathology progression. A growing body of evidence indicates that, under certain conditions, microglia are a vital source of important neurosupportive neurotrophins and anti-inflammatory cytokines, such as BDNF, IGF-1, and IL-4 [80,83,86,90,91]. Primarily M2a subtypes contributed to the repair of damaged tissue by expressing anti-inflammatory and neurotrophic factors [83]. Genes of the M2 phenotype are known to be essential for repair processes, and their high expression in our study might reflect an attempt at brain repair in response to VPA-induced abnormalities.

The limitation of these studies is that these experiments were performed exclusively on male young-adult offspring. For a long period of time it was believed that ASD is more frequent in males than in females. However, more recent works have raised some doubt and strengthened discussions on the topic of sex differences in autism and related disorders. The apparent underestimation of autism-related disorders in females is suggested to stem from different patterns of social deficiency masking by female patients [92,93]. Despite this, previous studies on the VPA model demonstrated both behavioural and neuroanatomical alterations in male offspring, while female offspring showed only marginal deficits [94,95,96]. Importantly, synaptic alterations were also more pronounced in male offspring, even if some changes were also present in females [94]. Therefore, we used only male offspring in this study, which was mainly oriented toward the pathological ultrastructural changes and biochemical alterations in the synapses. Our behavioural analysis confirmed the validity of the animal model used. The impact of gender on key VPA-evoked changes presented in this study should be analysed in detail in future research.

## 4. Materials and Methods

### 4.1. Ethical Statement

All experiments conducted with animals were approved by the Local Ethics Committee for Animal Experimentation in Warsaw, Poland (reference number 4/2014, 60/2015, 64/2015 and WAW2/148/2018), and were carried out in accordance with the EC Council Directive of November 24, 1986 (86/609/EEC) following the ARRIVE guidelines and guidelines published in the NIH Guide for the Care and Use of Laboratory Animals and the principles presented in the “Guidelines for the Use of Animals in Neuroscience Research” by the Society for Neuroscience. Efforts were made to minimise animal suffering and to reduce the number of animals used. All manipulations were performed gently and quickly to avoid stress-induced alterations.

### 4.2. Animals—In Vivo Model of ASD

Pregnant Wistar rats between 12 and 15 weeks of age weighing 210–250 g were supplied by the Animal House of the Mossakowski Medical Research Centre, Polish Academy of Sciences (Warsaw, Poland), which breeds small rodents according to the SPF standard. The animals were maintained under controlled conditions of temperature and humidity with a 12 h light/dark cycle. Procedures involving animals were carried out in strict accordance with international standards of animal care guidelines, and every effort was made to minimize suffering and the number of animals used. According to the study of Schneider and Przewlocki [31], an autism spectrum disorder (ASD) model was induced by a single intraperitoneal injection of VPA at a dose of 450 mg/kg of body weight on gestational day 12.5 (experimental group). Pregnant females from the control groups received a single intraperitoneal injection of solvent (sterile 0.9% NaCl). All pregnant dams were allowed free access to food and water and were kept in a room with a controlled temperature under an LD 12/12 regime to give birth. All dams were allowed to give birth and nurture their offspring under normal conditions. The day of birth was considered as postnatal day (PND) 1. On PND 7, each litter was equalised (random selection), and the number of pups was limited to 10 (both male and female). Offspring (males and females) stayed with their mothers and were fed by them until PND 22–23, and then the rat pups were separated and kept in groups of 3 or 4 in open polycarbonate cages in an enriched environment. To avoid the interference of hormonal disturbances/changes, only males were selected for further experimental procedures. Young-adult males were sacrificed at PND 58 by decapitation; their brains were quickly removed and dissected into two regions, the hippocampus and cerebral cortex, and then placed in liquid nitrogen. The samples were stored at −80 °C for further analysis. 

### 4.3. Behavioural Analysis

#### 4.3.1. Juvenile Isolation Test (ISO)

**Figure 11:**
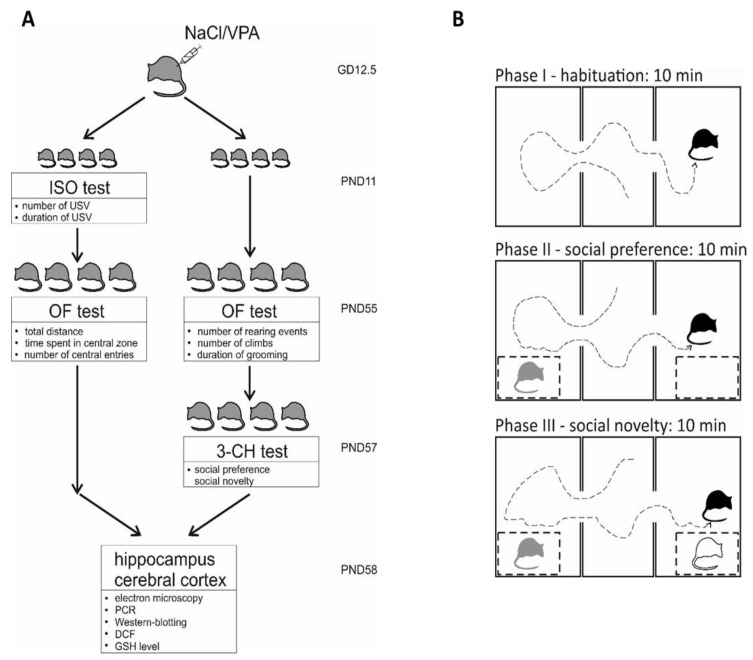
Experimental design. (**A**) Pregnant Wistar rats were injected at gestation day 12.5 (GD 12.5) with NaCl or VPA (450 mg/kg b.w.). One group of offspring rats (males and females) was subjected to an ISO test at postnatal day 11 (PND 11) and then to an Open-field test (males only) at PND 55. Other groups of offspring rats (males) was subjected to Open-field test at PND 55 and then to a 3-chamber test at PND 57. All offspring rats were euthanized at PND 58; tissue samples from the hippocampus and cerebral cortex were collected and used for biochemical, immunochemical, genetic, and microscopic analysis. (**B**) Design of the 3-chamber test. A detailed description is included in the behavioural analysis section.

The ISO test was performed according to the protocol described by Zieminska et al. [33]. The number of USV and their length were determined for the infant rats at PND 11 (Figure 11). Pups were taken individually from the breeding cage, placed in a glass container with fresh bedding, and lowered into a Styrofoam box (17 × 17 × 17 cm) with a CM16/CMPA ultrasound condenser microphone (Avisoft Bioacoustics, Glienicke/Nordbahn, Germany) located in the lid of the box. The recording session lasted for 5 min. USV were analysed using digital sound spectrographic analysis provided by an AviSoft Recorder USGH (Avisoft Bioacoustics, Glienicke/Nordbahn, Germany) and SASLab Pro (Avisoft Bioacoustics, Glienicke/Nordbahn, Germany).

#### 4.3.2. Open Field Test (OF)

The OF test was performed according to the protocol described by Milewski et al. [97] with modifications. This test provides a unique opportunity to systematically assess novel environment exploration, general locomotor activity, and an initial screen for anxiety-related behaviour in rodents. All tests were performed between 9:00 and 14:00 in a sound-isolated room with a constant illumination of ca. 25 lx at the level of the test chamber. Animals were habituated to the testing room for one week. Rats at 55 PND (Figure 11) were individually placed in the corner of the arena (dark grey PCV box 55 × 55 × 50 (h) cm), and the activity was recorded for 10 min with a video camera. The total distance moved, total time spent in the central zone, and number of entries to the central zone were measured using Ethovision XT 10 (Noldus Information Technology, Wageningen, The Netherlands). The duration of grooming and the number of climbs and rearings were scored manually by a blinded technician. After each trial, the test chamber was cleaned with 10% ethanol.

#### 4.3.3. Three-Chamber Sociability and Social Novelty Test

This test assesses cognition in the form of general sociability and interest in social novelty in rats. Rats normally prefer to spend more time with other rodents (sociability) and will investigate a novel intruder more than a familiar one (social novelty). At PND 57, rats were introduced to an apparatus (45 × 85 × 40 (h) cm) divided into three chambers (30 cm, 25 cm, and 30 cm) with 10 cm wide doors. The test consisted of three phases: I) 10 min habituation, II) 10 min social preference test, and III) 10 min social novelty test (Figure 11). In the habituation phase, the tested rat was placed in the central chamber and allowed to freely explore the whole empty apparatus. In phase II, the rat was confronted with 1) a cage containing an animal (an unknown rat of the same sex and weight) placed in one chamber and 2) an empty cage placed in the opposite chamber. In this phase, social preference was measured (social stimulus versus non-social stimulus). In phase III, the empty cage was replaced with a new cage containing a novel animal (an unknown rat of the same sex and weight). In this phase, social novelty was measured (a familiar social stimulus versus a novel social stimulus). The animal’s behaviour was recorded with a camera. The time spent in each chamber, the time spent on the exploration of each cage, and the time spent on direct interactions with the animal were measured by a blinded technician using the BehaView software (Warsaw, Poland).

### 4.4. Electron Microscopic Analysis of Brain Samples (TEM Analysis)

Rats at PND 58 were anaesthetized with a mixture of Ketamine and Xylazine (75 and 5 mg/kg, respectively, i.p.) perfused through the ascending aorta initially with 0.9% NaCl in 0.01 M sodium–potassium phosphate buffer (pH 7.4) and then with 2% paraformaldehyde and 2.5% glutaraldehyde in a 0.1 M cacodylate buffer, pH 7.4, at 20 °C (Sigma-Aldrich, St. Louis, MO, USA). Material for the ultrastructural studies was sampled from the frontal cerebral cortex and the CA1 region of the hippocampus for all rat groups. Specimens were fixed in an ice-cold fixative solution for 20 h and placed in a mixture of 1% OsO_4_ and 0.8% K_4_[Fe(CN)_6_]. After dehydration in a series of ethanol gradients, tissue specimens were embedded in epoxy resin (Epon 812). Ultra-thin sections (60 nm) were examined by transmission electron microscopy (JEM-1200EX, Jeol, Japan). Images were taken with a MORADA camera and the iTEM 1233 software. To assess the number of synaptic vesicles in the nerve terminals, electronograms under a magnification of 50,000 × were taken. The number of synaptic vesicles (SVs) was counted in 30 nerve endings in each animal from the control and experimental groups. The results are shown as the mean ± S.E.M from 4 animals for both the VPA and control groups.

### 4.5. Quantitative Real Time Polymerase Chain Reaction (qRT-PCR)

The quantitative analysis of the mRNA expression of the genes for synaptic proteins *Vamp1, Vamp2, Syp, Syn1, Syt1, Snap25, Stx1a, Stx1b, Dlg4, Shank2, Shank3, Nlgn1, Nlgn3, Ptgs2, Alox12,* and the pro-inflammatory cytokines (markers of M1 microglia activation) *Il1b, Il6, Tnf, Arg1, Chi3l1, Mrc1, Cd86, Fcgr1a, Tgfb1,* and *Sphk1* was performed by two-step reverse transcription PCR. RNA was isolated by using TRI-reagent according to the manufacturer’s protocol (Sigma-Aldrich, St. Louis, MO, USA). Digestion of the DNA contamination was performed by using DNase I according to the manufacturer’s protocol (Sigma-Aldrich, St. Louis, MO, USA). RNA quantity and quality were controlled by spectrophotometric analysis using a NanoDrop ND-1000 spectrophotometer (NanoDrop Technologies, Wilmington, DE, USA). Reverse transcription was performed using a High Capacity cDNA Reverse Transcription Kit according to the manufacturer’s protocol (Applied Biosystems, Foster City, CA, USA). Quantitative PCR was performed on an ABI PRISM 7500 apparatus using commercially available TaqMan Gene Expression Assays (Applied Biosystems, Foster City, CA, USA), according to the manufacturer’s protocol: *Vamp1* (Rn00565308_m1); *Vamp2* (Rn00360268_g1); *Syp* (Rn01528256_m1); *Syn1* (Rn00569468_m1); *Syt1* (Rn00436862_m1); *Snap25* (Rn00578534_m1); *Stx1a* (Rn00587278_m1); *Stx1b* (Rn01510167_m1); *Dlg4* (Rn00571479_m1); *Shank2* (Rn01479040_m1); *Shank3* (Rn00572344_m1); *Nlgn1* (Rn01642900_m1); *Nlgn3* (Rn01419002_m1); *Ptgs2* (Rn01483828_m1); *Alox12* (Rn01461081_m1); *Il1b* (Rn00580432_m1); *Il6* (Rn01410339_m1); *Tnf* (Rn01525859_g1); *Arg1* (Rn00567522_m1); *Chi3l1* (Rn01490608_m1); *Mrc1* (Rn01487342_m1); *Cd86* (Rn00571654_m1); *Fcgr1a* (Rn01762682_m1); *Tgfb1* (Rn00572010_m1); *Sphk1* (Rn00591307_m1); and *Actb* (Rn00667869_m1). *Actb* was analysed as a reference gene. The real-time conditions were as follows: 95 °C (15 s), 40 cycles at 95 °C (15 s), and 60 °C (1 min). Each sample was analysed in three technical replicates, and the mean Ct values were used for further analysis. The results were analysed by using the ∆∆Ct method.

### 4.6. Immunochemical Determination of Protein Levels (Western Blot Analysis)

The immunochemical analysis of the proteins level and phosphorylation status was performed by the Western blotting method under standard conditions. Tissue samples were homogenized, mixed with a Laemmli buffer, and denatured at 95 °C for 5 min. After standard SDS-PAGE separation, the proteins were “wet”-transferred to nitrocellulose membranes under standard conditions, and the proteins were detected by immunodetection with specific antibodies. The membranes were washed for 5 min in TBST (Tris-buffered saline with Tween 20 buffer: 100 mM Tris, 140 mM NaCl and 0.1% Tween 20, pH 7.6), and non-specific binding was blocked for 1 h at room temperature (RT) with 2% or 0.5% BSA in TBST or with 5% non-fat milk solution in TBST. Membranes were probed with the following primary antibodies: VAMP1/2 (1:500), Syp (1:750), Syn1 (1:500), p-Syn1 (1:500), Syt1 (1:1000), SNAP25 (1:1000), Stx1 (1:1000), PSD95 (1:1000), Shank2 (1:750), Shank3 (1:1000), Nlgn1 and Nlgn3 (1:200 and 1:500, respectively), and Iba-1 (1:1000) (Table 1). Glyceraldehyde 3-phosphate dehydrogenase (GAPDH, 1:50,000) and vinculin (VCL, 1:1000) were used as the loading controls. The membranes were washed three times in TBST, incubated for 60 min at RT with appropriate secondary antibodies (1:8000 anti-rabbit or 1:4000 anti-mouse IgG), and washed again 3 × in TBST. Antibodies were detected using the chemiluminescent reaction and an ECL reagent (Amersham Biosciences, Bath, UK) under standard conditions. After stripping, the immunolabelling of GAPDH and vinculin was performed as a loading control. Densitometric analysis was performed with the TotalLab software.

### 4.7. Determination of Glutathione Levels

The levels of total GSH content (i.e., both oxidized and reduced) and oxidized (GSSG) forms of glutathione, as well as the reduced forms of glutathione (GSH), were measured in the deproteinized homogenates of the control and VPA brain tissue using an enzymatic Glutathione Colorimetric Detection Kit according to the manufacturer’s instructions (Item No. 703002, Cayman Chemical, Ann Arbor, MI, USA). Tissues were homogenized in an ice-cold buffer (50 mM MES, pH 6-7; 1 mM EDTA) and centrifuged (10,000 × g, 15 min, 4 °C). The resulting supernatant was used to determine the protein content and was then deproteinized. The GSSG concentration was determined by a derivatization technique according to the manufacturer’s instructions. The reaction was initiated by adding a freshly prepared assay cocktail, and the change in absorbance was detected at 405 nm after 25 min. Reduced GSH was quantified using the following equation: GSH = Total glutathione − 2×GSSG.

### 4.8. Determination of Reactive Oxygen Species (ROS) Level

Measurement of the ROS level in tissue homogenates was performed by using a fluorescent probe 2′,7′-dichlorodihydrofluorescein diacetate (H_2_DCF-DA), which was deacetylated by cellular esterases to 2′,7′-dichlorodihydrofluorescein (H_2_DCF). H_2_DCF was oxidized by free radicals to the highly fluorescent compound, 2′,7′-dichlorofluorescein (DCF). To measure the ongoing generation of ROS in the rat brain, the tissue homogenate (1% in PBS) was incubated with 10 µM H_2_DCF-DA in the dark at 37 °C for 15 min (the phase of probe loading and deacetylation). Then, the analysis phase started. Incubation continued for 30 min, and DCF fluorescence was measured every 5 min using a microplate reader (TECAN Infinite M1000PRO, Grödig/Salzburg, Austria) at 488 nm excitation and 525 nm emission wavelengths. Each sample was analysed in triplicate. To confirm that the deacetylation of the probe was not a limiting factor of the reaction, each sample was incubated additionally in the presence of 10 µM FeCl_2_ (positive control). The results are presented as the relative increase in fluorescence.

### 4.9. Statistical Analysis of Biochemical and Behavioural Results

The results are expressed as the mean values ± S.E.M. Results more than twice the standard deviation away from the means (outliers) were excluded. Differences between the means were analysed using Student’s *t*-test for two groups or a one-way analysis of variance (ANOVA) with a Newman–Keuls post-hoc test among multiple groups; differences were considered significant at *p* < 0.05. The normality and equality of group variances were tested by a Shapiro–Wilk test. For the non-normal distribution of data, non-parametrical tests, such as the U-Mann–Whitney test were used. The statistical analysis was performed using Graph-Pad Prism version 8.0 (Graph Pad Software, San Diego, CA, USA).

## 5. Conclusions

**Figure 12:**
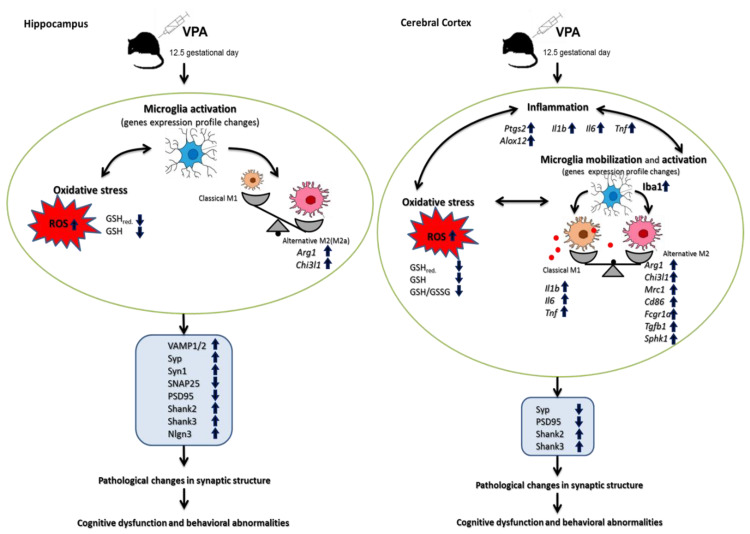
Schematic diagram showing the effect of prenatal exposure to VPA on the pathological changes in brains of young-adult male offspring. A single *i.p*. injection of VPA at 12.5 days of pregnancy-induced brain structure-specific defects in the expression of key pre- and postsynaptic proteins, consequently leading to pathological alterations in the structure of the synaptic endings (the hippocampus and cerebral cortex). In the hippocampus, the importance of oxidative stress and ROS generation in synaptic pathology is suggested. In the cerebral cortex, in addition to oxidative stress and ROS generation, neuroinflammation associated with microglia mobilization and M1 activation have also been proposed as potential triggers of a molecular cascade leading to synaptopathology. VPA induced long-lasting changes in microglia status in a brain structure-specific manner. In the cerebral cortex, both the pro-inflammatory M1 and the potentially beneficial recovery-promoting M2 phenotype were activated, while in the hippocampus, only M2 was stimulated.

Our study, for the first time, showed the drastic pathological changes in synaptic structure along with alterations in the levels of key pre- and postsynaptic proteins in both the hippocampus and cerebral cortex of young-adult offspring prenatally exposed to VPA. These observed abnormalities in synapses suggest synaptic dysfunction, which in consequence can lead to the impairment of neuronal connectivity and synaptic transmission and induce functional and cognitive impairments. Disruption of the synaptic structure and plasticity may represent a primary insult responsible for the autism-related behaviour in the offspring. The vulnerability of specific synaptic proteins to the epigenetic effects of VPA may highlight the potential mechanisms by which prenatal VPA exposure generates behavioural changes. In addition, our results highlighted the importance of oxidative stress, as well as the neuroinflammation associated with microglia activation (in a brain region-specific manner), as potential triggers of a molecular cascade leading to alterations of synaptic proteins and the structures of the synaptic endings (Figure 12). The obtained results may contribute to a better understanding of the synaptopathology underlying ASD.

## Figures and Tables

**Table 1:** Conditions used to perform the Western blot experiments.

**Primary Antibody**	**Brand/Cat #**	**Dilution**
Mouse anti-VAMP1/2	Santa Cruz Biotechnology sc-20039	1:500 5% milk in TBS-T 0.1%
Mouse anti-Syp	Santa Cruz Biotechnology sc-55507	1:750 TBS-T 0.1%
Mouse anti-Syn1	Santa Cruz Biotechnology sc-390867	1:500 TBS-T 0.1%
Rabbit anti-p-Syn1(Ser62/Ser67)	Santa Cruz Biotechnology sc-135709	1:500 TBS-T 0.1%
Rabbit anti-Syt1	Cell Signalling #3347	1:1000 5% BSA in TBS-T 0.1%
Rabbit anti-SNAP25	Cell Signalling #5309	1:1000 5% milk in TBS-T 0.1%
Mouse anti-Stx1	Santa Cruz Biotechnology sc-12736	1:1000 5% milk in TBS-T 0.1%
Mouse anti-PSD95	Santa Cruz Biotechnology sc-71935	1:1000 1% BSA in TBS-T 0.1%
Rabbit anti-Shank2	Cell Signalling #12218S	1:750 5% milk in TBS-T 0.1%
Mouse anti-Shank3	Abcam ab93607	1:1000 5% milk in TBS-T 0.1%
Mouse anti-Nlgn1	Santa Cruz Biotechnology sc-365110	1:200 5% milk in TBS-T 0.1%
Mouse anti-Nlgn3	Abcam ab186307	1:500 5% milk in TBS-T 0.1%
Rabbit anti-Iba1	Cell Signalling #17198	1:1000 TBS-T 0.1%
Rabbit anti-GAPDH	Sigma-Aldrich G9545-200UL	1:50,000 5% milk in TBS-T 0.1%
Rabbit anti-vinculin	Cell Signalling #13901	1:1000 5% milk in TBS-T 0.1%
**Secondary Antibody**	**Brand/Cat #**	**Dilution**
anti-mouse IgG	GE Healthcare VXA931V	1:4000 5% milk in TBS-T 0.1%
anti-rabbit IgG	Sigma-Aldrich A0545-1ML	1:8000 5% milk in TBS-T 0.1%

## Abbreviations

ASD	Autism Spectrum Disorder
VPA	Valproic acid
Nlgns	Neuroligins
PSD95	Post-synaptic density protein 95
Shank	SH3 and multiple ankyrin repeat domains protein
Syn	Synapsin
Nrnx	Neurexin
SNAP25	Synaptosomal-associated protein of 25 kDa
SNARE	Soluble N-ethylmaleimide-sensitive factor (NSF) attachment protein (SNAP) receptor
*v*-SNARE	Vesicle-associated membrane proteins
*t*-SNARE	Transmembrane-associated proteins
VAMP1/2	Synaptobrevins 1 and 2
Stx1	Syntaxin1
Syp	Synaptophysin
Syt	Synaptotagmin
ADHD	Attention deficit hyperactivity disorder
BPD	Bipolar disorder
SCH	Schizophrenia
MDD	Major depressive disorder
TEM	Transmission electron microscopy
SVs	Synaptic vesicles
PSD	Postsynaptic density
p-Syn1	Phospho-synapsin 1(Ser62/Ser67)
GSH	Glutathione
GSSG	Oxidised glutathione
IL-1β	Interleukin-1β
IL-6	Interleukin-6
TNF-α	Tumor necrosis factor α
Arg1	Arginase1
Chi3l1	Chitinase-3-like protein 1
Mrc1	Mannose receptor C-type 1
Cd86	CD86 molecule
Fcgr1α	Fc receptor subunits 1
Tgfb1	Transforming growth factor β
Sphk1	Sphingosine kinase 1
Alox12	Arachidonate 12-Lipoxygenase
LOX12	12-Lipoxygenase
Ptgs2	Prostaglandin-endoperoxide synthase 2
COX2	Cyclooxygenase 2
CNS	Central nervous system
NMDA	N-methyl-D-aspartate receptor
AMPA	α-amino-3-hydroxy-5-methyl-4-isoxazolepropionic acid receptor
AD	Alzheimer’s disease
PD	Parkinson’s disease
FXS	Fragile X syndrome
Iba 1	Ionised calcium binding adaptor molecule 1
NF-κB	Nuclear factor kappa β
BDNF	Brain-derived neurotrophic factor
IGF-1	Insulin like growth factor 1
